# Editorial: Safety and efficacy evaluation of electrical stimulation devices for neural modulation

**DOI:** 10.3389/fnins.2023.1143989

**Published:** 2023-02-07

**Authors:** Meijun Ye, Alexander G. Zestos, Cristin G. Welle, Guangying K. Wu, Xinyan Tracy Cui

**Affiliations:** ^1^Division of Biomedical Physics, Office of Science and Engineering Laboratories, Center for Devices and Radiological Health, Food and Drug Administration, Silver Spring, MD, United States; ^2^Department of Chemistry, American University, Washington, DC, United States; ^3^Department of Physiology and Biophysics, University of Colorado, Anschutz Medical Campus, Denver, CO, United States; ^4^Department of Bioengineering, University of Pittsburgh, Pittsburgh, PA, United States

**Keywords:** electrical stimulation, neuromodulation, safety, effectiveness, editorial

Neuromodulation through electrical stimulation has become increasingly promising for the treatment of neurological diseases, i.e., Parkinson's disease, depression, epilepsy, and for the restoration of sensory and motor functions. However, our understanding of the mechanisms underlying the effectiveness and safety of electrical stimulation is falling behind, which limits the possible therapeutic options for many novel stimulation devices or applications. This Research Topic collects studies focusing on unraveling the complicated interactions between stimulation devices and neural tissues. It intends to deepen our understanding of the biological and engineering mechanisms for safe, effective and reliable neural stimulation.

Concerns involving electrical stimulation through implantable devices include charge injection limits of electrodes, neural tissue damage, physiological side effects and device longevity. To evaluate the neural tissue damage, currently, the Shannon equation [*k* = log(D) + log(Q)] based on charge density (D, μcoulomb/phase·cm^2^) and charge per phase (Q, μcoulomb/phase) is widely cited to define the boundary between “safe” and “damaging” levels of electrical stimulation using macroelectrodes with a threshold at *k* = 1.85 (Shannon, 1992). For microelectrodes (electrodes with a geometrical surface area <2,000 μm^2^), a safety threshold of 4 nC (nanocoulomb) per phase was suggested (Cogan et al., 2016). Mounting evidence demonstrated that these thresholds restricted the device to suboptimal effectiveness. For example, multiple studies showed that the threshold for restoring motor or sensory function through intracortical stimulation ranges from 3 to 10 nC per phase and the sensation features can be finely tuned by adjusting the stimulation intensity up to 20 nC (Kim et al., 2015a; Flesher et al., 2016; Fernández et al., 2021). Though other stimulation parameters, such as frequency and duty cycle, need to be taken into consideration, the 4 nC safety threshold apparently cannot satisfy the need for effectiveness. Therefore, when novel devices or stimulation paradigms are developed, pre-clinical safety evaluation is usually required. Histology has been the gold standard for assessing neural damage, including neuronal and non-neuronal cells, in animals, while 2 photon imaging studies have begun to reveal the cellular response to electrical stimulation in real time (Histed et al., 2009; Kozai et al., 2016; Michelson et al., 2019). However, histology at a single time point and *in vivo* imaging may not be sufficient to capture subtle changes at the molecular level which can possibly lead to long-term deterioration. Whitsitt et al., proposed a novel method, spatial transcriptomics, to redefine electrical stimulation safety. In this study, they successfully demonstrated the concept by showing increased inflammatory and plasticity related genes surrounding a stimulating electrode. In addition, the spatial profiles of gene expression can be compared with histology. This emerging high-throughput method has the potential to discover new biomarkers of neurostimulation, moreover, the identified altered genes can serve as a therapeutic target to develop neuroprotective strategies for risk mitigation.

Though the primary target of electrical stimulation is to modulate neuronal activity, non-neuronal cells can be affected through neurochemical cascade. Williams et al. summarized the effect of different modalities of neurostimulation on non-neuronal cells from *in vitro* system to clinical studies. In this review, they found that studies have indicated that electrical stimulation can cause morphological and molecular changes of glia cells, including microglia, astrocytes, and oligodendrocytes. However, only a paucity of studies assessed the effect of electrical stimulation on endothelial cells and the blood-brain-barrier. These preliminary studies imply that electrical stimulation can possibly increase blood-brain-barrier permeability, but further investigation will be needed.

In addition to neuronal and non-neuronal tissue responses, the limitation on charge injection capacity is another significant safety factor involved in electrical stimulation. To overcome the safe injectable current limit, synchronous or interleaved stimulation paradigms were developed (Zaaimi et al., 2013; Kim et al., 2015b; Flesher et al., 2016). Capacitive decoupling has also been used for safer voltammetric measurements of neurotransmitters in the brain (Siegenthaler et al., 2020). Many prior research that investigated the use of simultaneous stimulation from multiple channels was based on arrays of microelectrodes located in a single cortical layer (Zaaimi et al., 2013; Kim et al., 2015b). Kunigk et al. expanded the research to electrode arrays that span multiple cortical layers. This is particularly important as multiple novel linear electrode arrays with very small geometrical surface area are under development and with intention to move to human testing (Luan et al., 2017; Musk and Neuralink, 2019). The small size of the electrodes will require the strict limitation of the charge delivered to avoid electrode damage. Kunigk et al.'s study showed that only half of the charge was needed per site to reach the detection thresholds when stimulating from two adjacent electrodes with 100 μm spacing at the same time. This finding will benefit the future development of neuroprosthetic devices by increasing the spatial resolution with smaller electrodes, while not posing additional risk to exceed the charge injection safety limit.

Vatsyayan and Dayeh's study further expanded our knowledge in the relationship between electrode design and the electrochemical safety limit. This report captures a systematic study from benchtop electrochemical measurement and analyses to the development and validation of a predictive model. They found that the charge injection capacity of the electrodes increases non-linearly with pulse width and electrode size. They developed a predictive equation to describe the dependence of cathodal excitation as a function of the electrode material, electrode size, and the amplitude and duration of the injected pulse. This study provides a valuable complement to Shannon's limit by taking into consideration the electrode material and surrounding media, and expanding it from macroelectrodes to microelectrodes.

When discussing the electrochemical safety of electrodes, changes in the surrounding environment and electrode properties over time need to be considered for safe chronic use. Frederick et al. evaluated the chronic stability of the voltage transients in 3,000 μm^2^ activated iridium oxide electrode in an accelerated manner both in saline and in the canine visual cortex. They noticed that in the benchtop testing, these electrodes remain stable in voltage transients after 540 h of continuous stimulation at 200 Hz and 16 nC, and no delamination was observed with scanning electron microscopy imaging. They also found that, for the *in vivo* environment, a 24-h continuous stimulation did not have a significant impact on the electrode properties compared to un-stimulated electrodes. This study supports the electrochemical safety for the chronic use of such electrodes of up to 16 nC per phase.

In addition to the aforementioned five articles tackling the fundamental questions related to safety and effectiveness of electrical stimulation devices, two more clinically relevant articles were also included in this Topic. Kullmann et al. reports on the biocompatibility of a Food and Drug Administration (FDA) cleared thin-film electrode device for intracranial stimulation and recording. This electrode is superior to the predicate in mechanical flexibility, which offers better conformity to brain surface convolutions. This is a great improvement in the electrode design as the mechanical stress applied to the brain is one of the risk factors involved in such implantable electrodes. Luo et al. also demonstrated a clinical success rate of up to 80% with pulsed radiofrequency stimulation of dorsal root ganglion in combination with transforaminal epidural steroid injection for the alleviation of chronic pain.

In summary, this topic collected seven cutting edge articles from benchtop to clinical studies, which ranged from addressing a specific issue with the use of electrical stimulation device, i.e., electrochemical safety, to introducing advanced characterization methods, such as spatial transcriptomics. The study of safety and effectiveness of electrical stimulation requires the knowledges and expertise from many disciplines including chemistry, physics, material science, electrical engineering, in addition to neuroscience and medicine. We hope this Research Topic serves as an open forum to attract more researchers across different disciplines into this research field, bring attentions to understudied areas, and stimulates discussions and collaborations, with the ultimate goal of accelerating new developments in the field of neuromodulation therapies.

## Author contributions

All authors listed have made a substantial, direct, and intellectual contribution to the work and approved it for publication.
